# Transhepatic embolization of jejunal variceal bleeding in a patient with liver cirrhosis: a case report

**DOI:** 10.1007/s00508-025-02560-4

**Published:** 2025-07-10

**Authors:** M. D. Rathenböck, G. Hagleitner, P. Pichler, K. Szabo, A. Moschen, A. Shamiyeh, F. Fellner

**Affiliations:** 1 Department of Gastroenterology and Hepatology, Kepler University Clinic, Medical Faculty, Johannes Kepler University, Linz, Austria; 2 Central Radiology Institute, Kepler University Clinic, Medical Faculty, Johannes Kepler University, Linz, Austria; 3 Department of General and Visceral Surgery, Kepler University Clinic, Medical Faculty, Johannes Kepler University, Linz, Austria

**Keywords:** Small bowel variceal bleeding, Transhepatic embolization, Liver cirrhosis, Jejunal varices, Portal hypertension

## Abstract

Jejunal variceal bleeding due to portal hypertension remains a rare but serious complication of decompensated liver cirrhosis. Potential treatment options include endoscopic, surgical or interventional management. We present a case report of a patient with intestinal blood loss due to jejunal variceal bleeding who was treated using interventional transhepatic embolization.

## Introduction

In patients with liver cirrhosis, the formation of portosystemic collaterals is a common occurrence. Frequent sites of formation of collaterals include the esophageal, the gastric and the rectal intestinal wall [1]. Small intestinal varices account for a minor proportion in the context of portal hypertension, with one of the rarest occurrences being the duodenal wall (1–3%). Small bowel varices are typically associated with a more progressed Child-Pugh class [1–4]. Varicose vessels in the small bowel can be categorized into vascular and nonvascular lesions with further classification into superficial and profound vessel abnormalities, which can be routinely assessed using video capsule endoscopy or double balloon enteroscopy [5–7].

With acute variceal bleeding representing one of the main severe and potentially life-threatening manifestations of decompensated cirrhotic liver disease, interventional angiography marks an increasingly more important role in the management of active bleeding and prevention of recurrent hemorrhagic episodes [8].

In a patient population with elevated risk of periinterventional bleeding, e.g., due to advanced liver cirrhosis, transhepatic or translienal embolization of variceal collateral vessels in portal hypertension is a viable option for patients unfit for surgery and with endoscopically inaccessible bleeding sources [9].

## Case presentation

A 64-year-old male patient presented to a rural hospital due to acute hematochezia and melena following a course of oral cortisone for a flare of gout arthritis. The past medical history was positive for alcohol-associated liver disease resulting in liver cirrhosis (Child-Pugh A) with low-grade esophageal varices without red spot signs and splenomegaly, alongside arterial hypertension. He had been admitted to hospital 10 years previously because of a perforated gastric ulcer necessitating emergency laparotomy.

At admission to the rural hospital, physical examination revealed a patient in acute distress and active hematochezia was observed. Vital parameters revealed hypotension with an elevated heart rate indicating a positive shock index due to bleeding. The remainder of the physical examination was unremarkable.

Laboratory data showed an initial hemoglobin level of 12.6 g/dL (normal range: 13.5–17.5 g/dL) which decreased to 9.6 g/dL within 24 h. The hematocrit was 35.1% (normal range: 40.0–53.0%). The patient showed leukocytosis at 14.2 G/L (normal range: 4.0–10.0 G/L) and an elevated C‑reactive protein (CRP) at 27.52 mg/L (normal range: 0.50–5.00 mg/L). BUN was also elevated at 27.0 mg/dL (normal range: 9.0–23.0 mg/dL). The remaining laboratory parameters were unremarkable.

Upon admission to the rural hospital, intravenous antibiotic therapy with ceftriaxone was initiated. Given the initial presentation of hematochezia without hematemesis, no vasopressive agents were administered, as variceal bleeding secondary to liver cirrhosis was not suspected clinically.

A contrast enhanced multiphasic computed tomography scan (CECT) revealed no evidence of active luminal bleeding into the colon and intestines. Findings consistent with liver cirrhosis, splenomegaly and small amounts of paracolic and subhepatal ascites were noted. The portal vein was partially thrombosed, portosystemic collaterals were observed. The superior mesenteric vein and its branches were dilated, with a venous drainage through the umbilical vein, both inferior epigastric veins and into both common femoral veins.

Within the first 24 h of hospitalization, the patient received eight units of red blood cells due to the vital parameters indicating refractory hemorrhagic shock and was transferred to the intensive care unit. After restoring hemodynamic stability through red blood cell transfusion, endoscopic evaluation was performed. Gastroscopy showed low-grade esophageal varices without active bleeding, no ulcers were identified in the upper gastrointestinal tract, including the proximal duodenum. A colonoscopy was discontinued at the sigmoid colon due to stool contamination, revealing blood clots in the distal colon.

Due to persistent unmanageable bleeding the patient was transferred to our institution. Gastroscopic and coloscopic re-evaluation found no active source of gastrointestinal bleeding. Due to staffing limitations during night shifts, enteroscopy was not available at the time of presentation. Hemoglobin levels continually decreased despite the administration of two additional units of red blood cells.

**Fig. 1:**
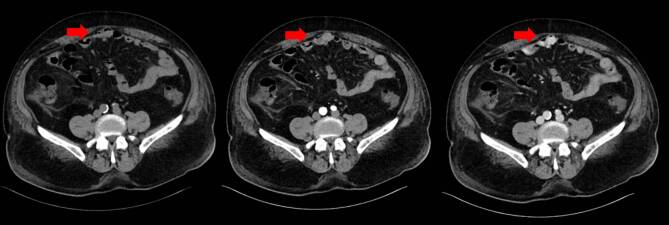
Abdominal computed tomography in transversal plane, showing the unenhanced (left), the arterial contrast enhanced (middle) and the venous sequence (right) of the examination. The venous sequence shows “pooling” as a sign of contrast extravasation into the small intestine, representing active bleeding into the jejunal lumen (*arrows*)

**Fig. 2:**
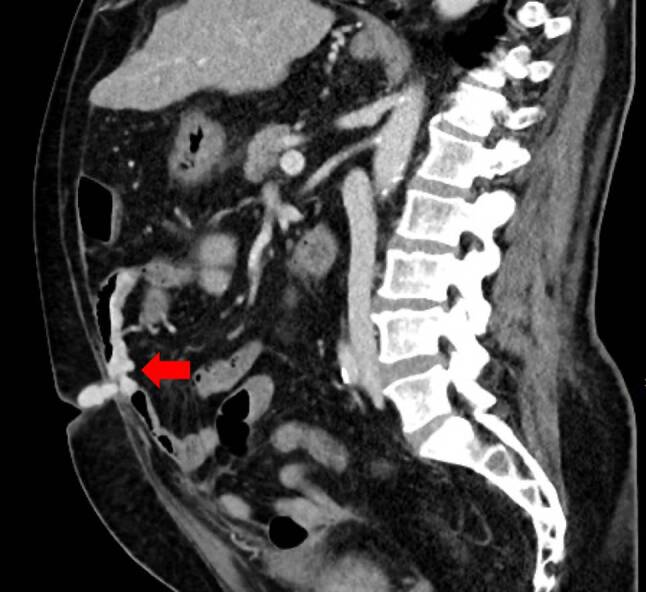
Venous “pooling” during contrast-enhances computed tomography as a sign of luminal bleeding into the jejunum in sagittal plane (*arrow*)

A repeated CECT scan (see Fig. 1, 2) showed active venous bleeding from small bowel varices in the jejunum, supplied by dilated veins in the mesenterium and the jejunal wall. The varices drained into a convoluted vascular sector in the periumbilical region, protruding into an umbilical hernia. The portal venous phase showed contrast extravasation into the intestinal lumen, representing “pooling” as a sign of venous bleeding into the small intestine. Other findings were consistent with prior imaging.

Given the increasing demand of red blood cell transfusion and the patient’s hemodynamic instability, he was classified unfit for surgery. The patient was prepared for the pursuit of angiographic intervention.

**Fig. 3:**
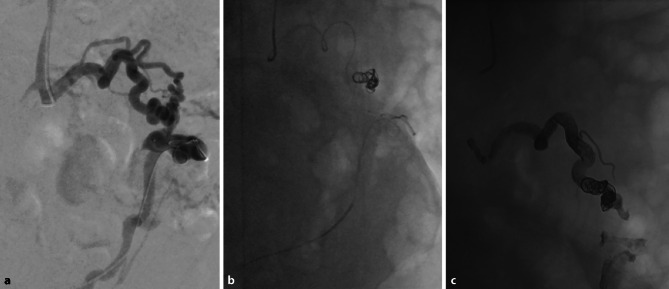
Tortuous veins forming a venous periumbilical convolute, as contrasted during interventional angiography. Catheters with coils in the proximal and distal segment of the convolute are shown (**a**). After successful selective embolisation from the transhepatic (upper end) and inferior epigastric (lower end) access, the detached coil is visible on x-ray (**b**). Onyx® embolus in situ with coil (**c**)

After establishing access to the right common femoral vein, the right inferior epigastric vein was cannulated. The bleeding site from the jejunal varices was angiographically confirmed after crossing the umbilical sector with a microcatheter (see Fig. 3). Due to vascular tortuosity the varices could not be crossed for frontdoor/backdoor embolization. Transhepatic puncture of the portal vein was performed. After cannulation of the superior mesenteric vein with another microcatheter, the feeding vessel of the jejunal varices was cannulated. Selective embolization was completed with two detachable coils (Interlock, Boston Scientific, Marlborough, MA, USA) and Onyx™ liquid embolic system (Medtronic, Dublin, Ireland) from the transhepatic access as well as Onyx™ (Medtronic, Dublin, Ireland) embolization from the transfemoral access. A final angiogram showed complete exclusion of the bleeding site.

After embolization no further hematochezia was observed and hemoglobin levels remained stable. Terlipressin perfusor treatment was started right after the establishment of the variceal jejunal bleeding. Anticoagulation with apixaban was initiated to address the partial portal vein thrombosis after stabilization and continuous clinical and laboratory recovery of the patient over the following post-interventional days. To reduce portal hypertension, treatment with the non-selective beta-blocker carvedilol was begun. Despite being offered psychological support to address alcohol intake, the patient declined.

Due to post-interventional transferal to a rural hospital, the patient was lost to a long-term follow-up.

## Discussion

In patients with cirrhotic liver disease and gastrointestinal bleeding or occult blood loss, small bowel variceal bleeding should be considered as a rare occurrence of variceal complication [10]. Ectopic varices account for 5% of variceal bleedings, and 17% of those occur in the jejunum [10]. Acute hematochezia due to small bowel variceal bleeding can lead to life-threatening hemodynamic instability.

Management of small bowel bleeding should involve a multidisciplinary team including gastroenterologists, interventional radiologists and surgeons [10]. Therapeutic decisions should be guided by the underlying cause, location and extent of the hemorrhage, alongside the patient’s clinical condition and the local expertise available [10]. Endoscopic interventions may include endoscopic band ligation and endoscopic injection sclerotherapy [10]. In the case presented, an endoscopic approach was not feasible due to the inaccessibility of the bleeding site. Surgical options, such as involving resection of the affected segment or the creation of mesocaval or portocaval shunts and surgical devascularization were considered; however, given the patient’s poor surgical candidacy and the significant perioperative morbidity and mortality associated with such interventions, surgery was not appropriate.

In contrast to surgery performed with the patient under general anesthesia, endovascular procedures, typically conducted with local anesthesia, result in a minimized hemodynamic burden on the cardiovascular system. Viable endovascular strategies include the creation of a transjugular intrahepatic portosystemic shunt (TIPS) with or without embolization, balloon-occluded retrograde transvenous obliteration (B-RTO) and percutaneous embolization. While the percutaneous transhepatic approach demonstrates a high success rate of nearly 80% for rapid hemorrhage control, rebleeding rates can reach up to 65%. Alternative access routes may include translienal puncture or ultrasound-guided direct puncture of the varices. In this case, emergency TIPS was not indicated due to the initially unclear etiology of the gastrointestinal bleeding source: at the time of diagnosis of the variceal bleeding, the patient had already received coiling during angiographic intervention. Postprocedurally, the evaluation regarding a pre-emptive TIPS was not performed due to the patient’s low Child-Pugh score and due to the early re-transferral of the patient.

A multidisciplinary decision was made to proceed with percutaneous transfemoral and transhepatic embolization. Although randomized studies are lacking, the literature supports the diverse approaches in specific clinical scenarios. Thus, a multidisciplinary approach is essential, ensuring that therapeutic decisions are tailored to each case, considering the underlying cause, location and extent of the hemorrhage as well as the patient’s clinical status and the local expertise available.

Based on a review of the literature, only a limited number of cases of jejunal variceal bleeding secondary to liver cirrhosis are reported. Park et al. [11] presented a case of a 55-year-old female patient with a history of overt gastrointestinal bleeding, confirmed by capsule endoscopy and treated by interventional embolization. Similarly, Lee et al. [12] described a case of a patient with jejunal variceal bleeding with a history of liver cirrhosis and portal vein thrombosis, also managed with interventional embolization.

## Conclusion

Interventional angiographic embolization of jejunal bleeding is pivotal in managing acute variceal bleeding in patients with liver cirrhosis. For those who are not suitable candidates for surgery, interventional angiography represents a potentially life-saving approach to address the source of bleeding. Further research is needed to compare the outcomes of interventional embolisation versus surgical treatment, emphasising periprocedural risks, clinician experience, and post-procedural care.
